# Retrograde Tibiopedal Access as a Bail-Out Procedure for Endovascular Intervention Complications

**DOI:** 10.1155/2016/7519748

**Published:** 2016-10-10

**Authors:** Ahmed Amro, Alaa Gabi, Adee Elhamdani, Naveed Iqbal, Mehiar El-Hamdani

**Affiliations:** Joan C. Edwards School of Medicine, Marshall University, Huntington, WV, USA

## Abstract

*Introduction*. Retrograde pedal access has been well described in the literature as a secondary approach for limb salvage in critical limb ischemia (CLI) patients. In this manuscript we are presenting a case where retrograde tibiopedal access has been used as a bail-out procedure for the management of superficial femoral artery (SFA) intervention complications.* Procedure/Technique*. After development of a perforation while trying to cross the totally occluded mid SFA using the conventional CFA access, we were able to cross the mid SFA lesion after accessing the posterior tibial artery in a retrograde fashion and delivered a self-expanding stent which created a flap that sealed the perforation without the need for covered stent.* Conclusion*. Retrograde tibiopedal access is a safe and effective approach for delivery of stents from the distal approach and so can be used as a bail-out technique for SFA perforation.

## 1. Introduction

Retrograde pedal access has been well described in the literature as a secondary approach for limb salvage either in critical limb ischemia (CLI) patients after failure of antegrade femoral approach or in patients who are not candidates for the antegrade approach. In this manuscript we are presenting a case where retrograde tibiopedal access has been used as a bail-out procedure for the management of superficial femoral artery (SFA) intervention complications.

## 2. Case Presentation

A 76-year-old female with history of diabetes mellitus, hypertension, coronary artery disease, and smoking was complaining of intermittent severe claudication in her left leg with a Rutherford classification of 3. Left leg ABI was 0.61.

## 3. Procedure/Technique

**Figure 1:**
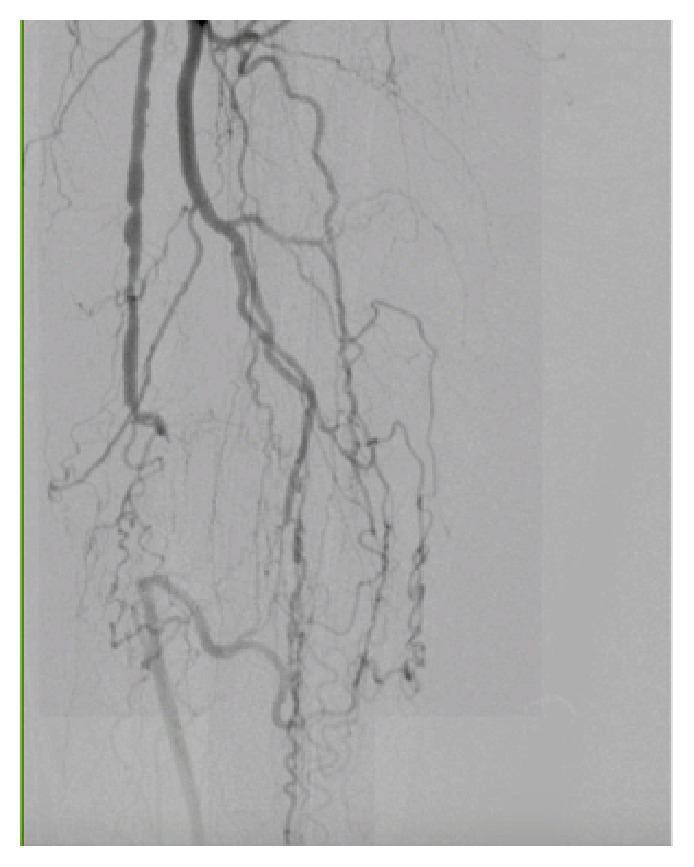
Totally occluded SFA.

**Figure 2:**
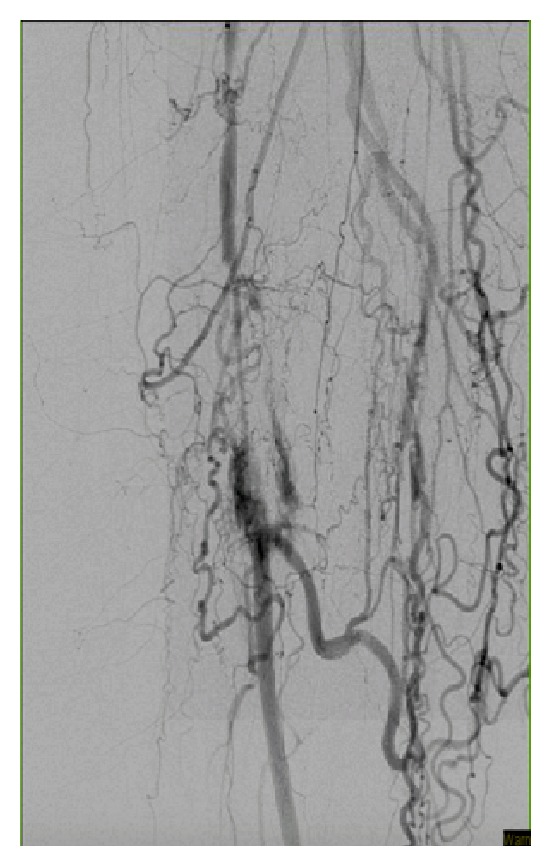
SFA perforated after Glidewire insertion in an attempt to cross the lesion.

**Figure 3:**
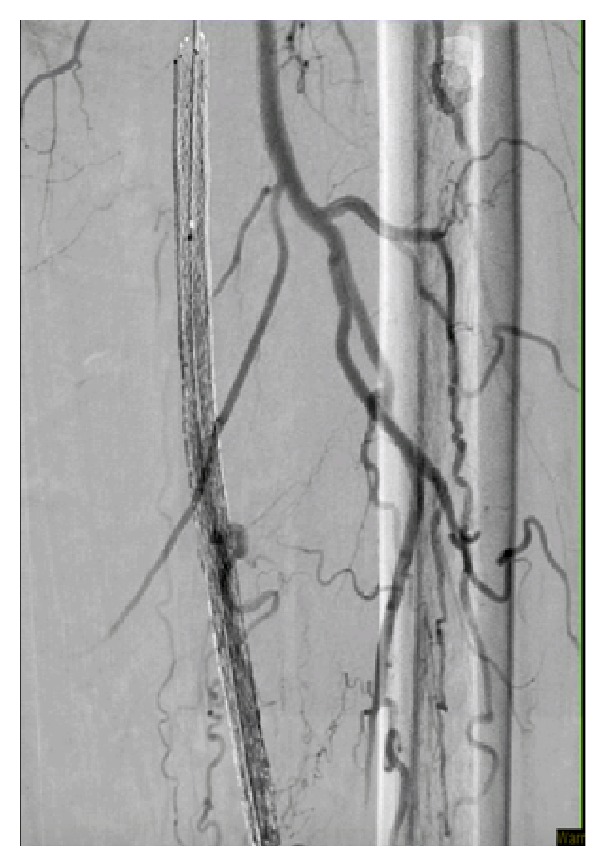
A retrograde self-expanding stent.

**Figure 4:**
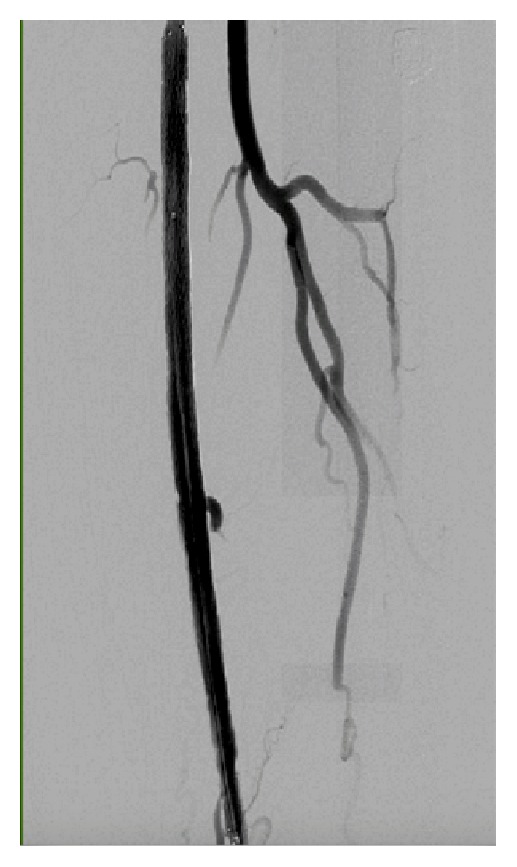
Complete resolution of perforation and a large collateral branch that is jailed by the stent.

Preintervention angiogram showed totally occluded mid SFA that reconstitutes to the distal SFA, which was patent as shown in Figure 1. While trying to cross the totally occluded mid SFA using the conventional CFA access, a perforation developed exactly just before the lesion as shown Figure 2. The wire was exchanged with Glidewire Gold in an attempt to cross the lesion without success in crossing the lesion. So the catheter was removed and EverCross balloon was inserted and inflated proximal to the perforation at 2 atmospheres in an attempt to control the bleeding by using balloon tamponade across the origin of the bleeding site. Access was then obtained through the left posterior tibial artery using an ultrasound. A 6 French glidesheath was inserted that was followed by antispasmodic cocktail of intra-arterial nitroglycerin and verapamil. Then a Gold-tip wire was inserted through the pedal sheath along with a NaviCross catheter that was used successfully to cross the totally occluded mid SFA. After that the catheter was removed and a Protégé EverFlex self-expanding stent was inserted and deployed in the mid SFA as shown in Figure 3 which created a flap that sealed the perforation without the need for a covered stent. To minimize the risk for access vessel thrombosis, the pedal sheath and deployment system was removed as soon as the stent was deployed and the perforation was sealed. Hemostasis was achieved by holding pressure manually for 10 minutes at the pedal access site. After the procedure an angiogram was obtained which revealed resolution of the perforation as shown in Figure 4.

## 4. Discussion

Peripheral arterial occlusive disease (PAD) is a common and debilitating disease that affects an estimated 10–12 million people in the United States [1]. Endovascular intervention (EI) is being routinely used as a primary approach for the treatment of obstructive PAD, which is often advocated based on lower procedure risk. Traditionally, the common femoral artery is primarily chosen for initial access site. Classic complications of antegrade femoral approach are retroperitoneal bleed, hematoma, perforations, pseudoaneurysm, and AV fistula. In this paper we are describing the retrograde tibiopedal access approach as a bail-out approach for the management of CFA approach complications while treating PAD involving the superficial femoral artery. Since it was originally described by Spinosa et al. and Botti Jr. et al., retrograde tibiopedal access has been well described in the literature as an effective, safe, and feasible approach with high technical success rate and relatively low procedural complication rates [2, 3]. However, it is currently considered as an alternative endovascular approach for limb salvage in patients with CLI after failed antegrade femoral artery access approach or for patients who are not candidates for antegrade approach. The advantages of tibiopedal access in general have been well described in the literature; it has significantly improved the success of revascularization in CLI lesions involving the infrapopliteal anatomy, with favorable outcomes technically, as well as lower 30-day mortality and higher rates of freedom from major adverse limb events and limb salvage up to one year in some reports [4, 5]. Another major advantage of the retrograde tibiopedal access is that it allows quick therapy and short procedure time with less observation time in the hospital. It is the preferred access in those patients with hostile groins, infected groins, morbid obesity, or other comorbidities. In this case we found that a quick tibiopedal access was safe, practical, and timely critical in managing superficial artery perforation while maintaining balloon tamponade above the lesion; hence, traditional tamponade across the lesion was not possible due to the site of the perforation which was exactly just before the lesion. Another point to bring to attention is that the retrograde tibiopedal approach allowed us to create a retrograde flap, which completely sealed the perforation using self-expandable noncovered stent in simple perforation. One of the most important successful factors in tibiopedal retrograde approach is adequate knowledge and training, as it is an evolving approach. We are still in the learning curve with more evolving ideas and techniques to come.

## 5. Conclusion

Retrograde tibiopedal access is a safe and effective approach for delivery of stents from the distal approach and so can be used as a bail-out technique for SFA perforation. Physicians who are involved in peripheral intervention will require having a full understanding of this approach and acquiring the necessary skills to perform it safely and effectively.
